# Supplementary material for: Resolving the full spectrum of human genome variation using Linked-Reads
Source: Genome Res. 2019 Apr;29(4):635–45. doi: 10.1101/gr.234443.118 (PMC6442396; doi:10.1101/gr.234443.118)
Supplement: Supplemental Material [file supp_gr.234443.118_Supplemental_longranger-2.2.zip › longranger-2.2.tar.bz2!/lr2.2-public/lib/stan/cmdstan/stan/doc/api/html/classstan_1_1gm_1_1function__signatures.html]

Stan: stan::gm::function\_signatures Class Reference


|  |  |
| --- | --- |
|  | Stan  2.5.0  probability, sampling & optimization |


- Main Page
- Related Pages
- Namespaces
- Classes
- Files

- Class List
- Class Index
- Class Hierarchy
- Class Members

All Classes Namespaces Files Functions Variables Typedefs Enumerations Enumerator Friends Macros Pages

- stan
- gm
- function\_signatures

Public Member Functions |
Static Public Member Functions |
List of all members

stan::gm::function\_signatures Class Reference

`#include <ast.hpp>`

|  |  |
| --- | --- |
| Public Member Functions | |
| void | set\_user\_defined (const std::pair< std::string, function\_signature\_t > &name\_sig) |
|  | |
| bool | is\_user\_defined (const std::pair< std::string, function\_signature\_t > &name\_sig) |
|  | |
| void | add (const std::string &name, const expr\_type &result\_type, const std::vector< expr\_type > &arg\_types) |
|  | |
| void | add (const std::string &name, const expr\_type &result\_type) |
|  | |
| void | add (const std::string &name, const expr\_type &result\_type, const expr\_type &arg\_type) |
|  | |
| void | add (const std::string &name, const expr\_type &result\_type, const expr\_type &arg\_type1, const expr\_type &arg\_type2) |
|  | |
| void | add (const std::string &name, const expr\_type &result\_type, const expr\_type &arg\_type1, const expr\_type &arg\_type2, const expr\_type &arg\_type3) |
|  | |
| void | add (const std::string &name, const expr\_type &result\_type, const expr\_type &arg\_type1, const expr\_type &arg\_type2, const expr\_type &arg\_type3, const expr\_type &arg\_type4) |
|  | |
| void | add (const std::string &name, const expr\_type &result\_type, const expr\_type &arg\_type1, const expr\_type &arg\_type2, const expr\_type &arg\_type3, const expr\_type &arg\_type4, const expr\_type &arg\_type5) |
|  | |
| void | add (const std::string &name, const expr\_type &result\_type, const expr\_type &arg\_type1, const expr\_type &arg\_type2, const expr\_type &arg\_type3, const expr\_type &arg\_type4, const expr\_type &arg\_type5, const expr\_type &arg\_type6) |
|  | |
| void | add (const std::string &name, const expr\_type &result\_type, const expr\_type &arg\_type1, const expr\_type &arg\_type2, const expr\_type &arg\_type3, const expr\_type &arg\_type4, const expr\_type &arg\_type5, const expr\_type &arg\_type6, const expr\_type &arg\_type7) |
|  | |
| void | add\_nullary (const ::std::string &name) |
|  | |
| void | add\_unary (const ::std::string &name) |
|  | |
| void | add\_binary (const ::std::string &name) |
|  | |
| void | add\_ternary (const ::std::string &name) |
|  | |
| void | add\_quaternary (const ::std::string &name) |
|  | |
| int | num\_promotions (const std::vector< expr\_type > &call\_args, const std::vector< expr\_type > &sig\_args) |
|  | |
| expr\_type | get\_result\_type (const std::string &name, const std::vector< expr\_type > &args, std::ostream &error\_msgs) |
|  | |
| int | get\_signature\_matches (const std::string &name, const std::vector< expr\_type > &args, function\_signature\_t &signature) |
|  | |
| bool | is\_defined (const std::string &name, const function\_signature\_t &sig) |
|  | |
| std::set< std::string > | key\_set () const |
|  | |

|  |  |
| --- | --- |
| Static Public Member Functions | |
| static function\_signatures & | instance () |
|  | |
| static void | reset\_sigs () |
|  | |

## Detailed Description

Definition at line 106 of file ast.hpp.

## Member Function Documentation

|  |  |  |  |
| --- | --- | --- | --- |
| void stan::gm::function\_signatures::add | ( | const std::string & | *name*, |
|  |  | const expr\_type & | *result\_type*, |
|  |  | const std::vector< expr\_type > & | *arg\_types* |
|  | ) |  |  |

Definition at line 171 of file ast\_def.cpp.

|  |  |  |  |
| --- | --- | --- | --- |
| void stan::gm::function\_signatures::add | ( | const std::string & | *name*, |
|  |  | const expr\_type & | *result\_type* |
|  | ) |  |  |

Definition at line 177 of file ast\_def.cpp.

|  |  |  |  |
| --- | --- | --- | --- |
| void stan::gm::function\_signatures::add | ( | const std::string & | *name*, |
|  |  | const expr\_type & | *result\_type*, |
|  |  | const expr\_type & | *arg\_type* |
|  | ) |  |  |

Definition at line 182 of file ast\_def.cpp.

|  |  |  |  |
| --- | --- | --- | --- |
| void stan::gm::function\_signatures::add | ( | const std::string & | *name*, |
|  |  | const expr\_type & | *result\_type*, |
|  |  | const expr\_type & | *arg\_type1*, |
|  |  | const expr\_type & | *arg\_type2* |
|  | ) |  |  |

Definition at line 189 of file ast\_def.cpp.

|  |  |  |  |
| --- | --- | --- | --- |
| void stan::gm::function\_signatures::add | ( | const std::string & | *name*, |
|  |  | const expr\_type & | *result\_type*, |
|  |  | const expr\_type & | *arg\_type1*, |
|  |  | const expr\_type & | *arg\_type2*, |
|  |  | const expr\_type & | *arg\_type3* |
|  | ) |  |  |

Definition at line 198 of file ast\_def.cpp.

|  |  |  |  |
| --- | --- | --- | --- |
| void stan::gm::function\_signatures::add | ( | const std::string & | *name*, |
|  |  | const expr\_type & | *result\_type*, |
|  |  | const expr\_type & | *arg\_type1*, |
|  |  | const expr\_type & | *arg\_type2*, |
|  |  | const expr\_type & | *arg\_type3*, |
|  |  | const expr\_type & | *arg\_type4* |
|  | ) |  |  |

Definition at line 209 of file ast\_def.cpp.

|  |  |  |  |
| --- | --- | --- | --- |
| void stan::gm::function\_signatures::add | ( | const std::string & | *name*, |
|  |  | const expr\_type & | *result\_type*, |
|  |  | const expr\_type & | *arg\_type1*, |
|  |  | const expr\_type & | *arg\_type2*, |
|  |  | const expr\_type & | *arg\_type3*, |
|  |  | const expr\_type & | *arg\_type4*, |
|  |  | const expr\_type & | *arg\_type5* |
|  | ) |  |  |

Definition at line 222 of file ast\_def.cpp.

|  |  |  |  |
| --- | --- | --- | --- |
| void stan::gm::function\_signatures::add | ( | const std::string & | *name*, |
|  |  | const expr\_type & | *result\_type*, |
|  |  | const expr\_type & | *arg\_type1*, |
|  |  | const expr\_type & | *arg\_type2*, |
|  |  | const expr\_type & | *arg\_type3*, |
|  |  | const expr\_type & | *arg\_type4*, |
|  |  | const expr\_type & | *arg\_type5*, |
|  |  | const expr\_type & | *arg\_type6* |
|  | ) |  |  |

Definition at line 237 of file ast\_def.cpp.

|  |  |  |  |
| --- | --- | --- | --- |
| void stan::gm::function\_signatures::add | ( | const std::string & | *name*, |
|  |  | const expr\_type & | *result\_type*, |
|  |  | const expr\_type & | *arg\_type1*, |
|  |  | const expr\_type & | *arg\_type2*, |
|  |  | const expr\_type & | *arg\_type3*, |
|  |  | const expr\_type & | *arg\_type4*, |
|  |  | const expr\_type & | *arg\_type5*, |
|  |  | const expr\_type & | *arg\_type6*, |
|  |  | const expr\_type & | *arg\_type7* |
|  | ) |  |  |

Definition at line 254 of file ast\_def.cpp.

|  |  |  |  |  |  |
| --- | --- | --- | --- | --- | --- |
| void stan::gm::function\_signatures::add\_binary | ( | const ::std::string & | *name* | ) |  |

Definition at line 279 of file ast\_def.cpp.

|  |  |  |  |  |  |
| --- | --- | --- | --- | --- | --- |
| void stan::gm::function\_signatures::add\_nullary | ( | const ::std::string & | *name* | ) |  |

Definition at line 273 of file ast\_def.cpp.

|  |  |  |  |  |  |
| --- | --- | --- | --- | --- | --- |
| void stan::gm::function\_signatures::add\_quaternary | ( | const ::std::string & | *name* | ) |  |

Definition at line 285 of file ast\_def.cpp.

|  |  |  |  |  |  |
| --- | --- | --- | --- | --- | --- |
| void stan::gm::function\_signatures::add\_ternary | ( | const ::std::string & | *name* | ) |  |

Definition at line 282 of file ast\_def.cpp.

|  |  |  |  |  |  |
| --- | --- | --- | --- | --- | --- |
| void stan::gm::function\_signatures::add\_unary | ( | const ::std::string & | *name* | ) |  |

Definition at line 276 of file ast\_def.cpp.

|  |  |  |  |
| --- | --- | --- | --- |
| expr\_type stan::gm::function\_signatures::get\_result\_type | ( | const std::string & | *name*, |
|  |  | const std::vector< expr\_type > & | *args*, |
|  |  | std::ostream & | *error\_msgs* |
|  | ) |  |  |

Definition at line 329 of file ast\_def.cpp.

|  |  |  |  |
| --- | --- | --- | --- |
| int stan::gm::function\_signatures::get\_signature\_matches | ( | const std::string & | *name*, |
|  |  | const std::vector< expr\_type > & | *args*, |
|  |  | function\_signature\_t & | *signature* |
|  | ) |  |  |

Definition at line 307 of file ast\_def.cpp.

|  |  |  |  |  |
| --- | --- | --- | --- | --- |
| function\_signatures & stan::gm::function\_signatures::instance | ( |  | ) |  |

static

Definition at line 143 of file ast\_def.cpp.

|  |  |  |  |
| --- | --- | --- | --- |
| bool stan::gm::function\_signatures::is\_defined | ( | const std::string & | *name*, |
|  |  | const function\_signature\_t & | *sig* |
|  | ) |  |  |

Definition at line 163 of file ast\_def.cpp.

|  |  |  |  |  |  |
| --- | --- | --- | --- | --- | --- |
| bool stan::gm::function\_signatures::is\_user\_defined | ( | const std::pair< std::string, function\_signature\_t > & | *name\_sig* | ) |  |

Definition at line 157 of file ast\_def.cpp.

|  |  |  |  |  |
| --- | --- | --- | --- | --- |
| std::set< std::string > stan::gm::function\_signatures::key\_set | ( |  | ) | const |

Definition at line 380 of file ast\_def.cpp.

|  |  |  |  |
| --- | --- | --- | --- |
| int stan::gm::function\_signatures::num\_promotions | ( | const std::vector< expr\_type > & | *call\_args*, |
|  |  | const std::vector< expr\_type > & | *sig\_args* |
|  | ) |  |  |

Definition at line 288 of file ast\_def.cpp.

|  |  |  |  |  |
| --- | --- | --- | --- | --- |
| void stan::gm::function\_signatures::reset\_sigs | ( |  | ) |  |

static

Definition at line 138 of file ast\_def.cpp.

|  |  |  |  |  |  |
| --- | --- | --- | --- | --- | --- |
| void stan::gm::function\_signatures::set\_user\_defined | ( | const std::pair< std::string, function\_signature\_t > & | *name\_sig* | ) |  |

Definition at line 150 of file ast\_def.cpp.

---

The documentation for this class was generated from the following files:

- src/stan/gm/ast.hpp
- src/stan/gm/ast\_def.cpp


---

[ Stan Home Page ]

*© 2011–2014,
Stan Development Team.*
